# Self-Report and Brain Indicators of Impaired Emotion Regulation in the Broad Autism Spectrum

**DOI:** 10.1007/s10803-017-3138-9

**Published:** 2017-04-26

**Authors:** Kristel De Groot, Jan W. Van Strien

**Affiliations:** Faculty of Social Sciences, Erasmus University Rotterdam, Burgemeester Oudlaan 50, 3062 PA Rotterdam, The Netherlands

**Keywords:** Autism spectrum disorder (ASD), Emotion regulation, Late positive potential (LPP), Reappraisal, Autism spectrum hypothesis

## Abstract

Although not used as a diagnostic criterion, impaired emotion regulation is frequently observed in autism. The present study examined self-reported use of emotion regulation strategies in individuals scoring low or high on autistic traits. In addition, the late positive potential, which is sensitive to emotional arousal, was used to examine the effect of one strategy, reappraisal. Reporting more autistic traits was related to using more maladaptive and fewer adaptive emotion regulation strategies. Across both groups, no attenuation of the late positive potential during downregulation of unpleasant pictures was found, possibly because of the used valence-changing reappraisal operationalisation. Hence, although self-report indicated impaired emotion regulation in individuals high on autistic traits, electrophysiological findings could not confirm this.

## Introduction

Emotion regulation (ER) encompasses the capacity to modulate or control the duration, latency, magnitude, and valence of one’s emotional reactions in order to behave adaptively and thereby meet situational demands (for a review, see Gross 2002). ER does therefore not only cover the ability to decrease negative emotions, but pertains to increasing, maintaining, or decreasing positive or negative emotions according to the situation one is in (Koole 2009). Although many ER strategies exist, research has mostly focussed on two: reappraisal and suppression. Reappraisal is used early on in the emotional process, and encompasses changing the way one thinks about emotional stimuli or emotional events. Suppression comes later in the emotional process, and covers changing one’s behavioural response to these stimuli or events (Cutuli 2014; Gross 2002). Thus, reappraisal focusses on reinterpretation, while suppression is more focussed on hiding or inhibition. The consequences of using reappraisal are judged as healthier than the consequences of suppression. While reappraisal changes both the emotional experience and the behavioural expression of that experience, suppression fails to impact the former (Cutuli 2014; John and Gross 2004). This has several consequences. First, while reappraisal decreases negative emotion and increases positive emotion, suppression fails to reduce negative emotion, and even leads to increased physiological activation (Cutuli 2014; Gross 1998, 2002; John and Gross 2004). In addition, because suppression comes relatively late in the emotional process, it takes up more cognitive resources than reappraisal, thereby impairing memory (Cutuli 2014; Gross 2002; John and Gross 2004). These depleted cognitive resources can, in turn, impair social functioning, since individuals who suppress emotions fail to absorb information needed for an adequate social response (Cutuli 2014; John and Gross 2004). Finally, reappraisal and suppression have different effects on psychological well-being. While the use of reappraisal is related to higher life-satisfaction, increased optimism, and better self-esteem, the use of suppression is linked to higher levels of depression (John and Gross 2004). In general, difficulty in controlling negative emotional responses is linked to problems in mood and anxiety (Campbell-Sills et al. 2006; Hofmann 2014). On the other hand, increased control over positive and negative emotions has been associated with enhanced positive (up-regulated) and reduced negative (down-regulated) feelings, respectively (Gross et al. 1997).

Focussing on more chronic ER impairments, studies show that persistent inability to successfully regulate emotions is implicated in the development and maintenance of many psychiatric problems (Aldao et al. 2010; Campbell-Sills et al. 2006; Koole 2009). Moreover, ER related characteristics are part of the diagnostic criteria of several mental disorders, most notably mood disorders, anxiety disorders, and some cluster B personality disorders such as borderline personality disorder and histrionic personality disorder (American Psychiatric Association 2013). However, they have mostly been ignored in disorders that do not include ER as a formal diagnostic criterion, but that often show ER disturbances nonetheless (Mazefsky 2015). One such disorder is autism spectrum disorder (ASD), a pervasive neurodevelopmental syndrome that includes social communicational deficits, restricted and repetitive behaviour, preference for sameness and routines, and sensory abnormalities (American Psychiatric Association 2013). While someone does not need to show impaired ER to be diagnosed with ASD, both clinicians and family members have noticed emotional deficits in individuals afflicted with the disorder, including irritability, amplified emotional responses, poor emotional control (‘meltdowns’ or ‘outbursts’), and poor stress management (Bauminger et al. 2010; Mazefsky 2015; Mazefsky et al. 2013).

These observations are supported by several studies showing that individuals with ASD indeed have poor ER capacity. Children and adolescents with ASD rely, compared to typically developing (TD) individuals, more on maladaptive than adaptive ER strategies (Samson et al. 2014a). Although the level of reactivity to negative real world scenarios did not differ, individuals with ASD used less cognitive reappraisal and made more use of suppression. The difficulty that individuals with ASD exhibited in generating cognitive reappraisal even persisted when the technique was explained to them and when they were encouraged to use the strategy. The other examined ER strategies, avoidance, distraction, problem solving, relaxation, and venting, did not differ between groups. These findings were partially supported by parent-reports and daily diaries from children and adolescents with ASD, who showed less use of adaptive and more use of maladaptive ER strategies (Samson et al. 2015b). Averaging across multiple emotions, especially the frequency and efficacy of using acceptance, cognitive reappraisal, distraction, and problem-solving differed between the ASD and TD group. Focussing only on reappraisal and suppression, Samson et al. (2012) found that adults with ASD showed a deviant ER pattern as well: individuals with ASD had difficulty in consciously down-reinterpreting the meaning of negative and up-regulating the meaning of positive emotional responses (together called cognitive reappraisal), and made more use of suppression.

Taking the deviant ER pattern found in individuals with ASD a step further, several studies have linked emotion dysregulation to comorbid pathological outcomes. Mazefsky et al. (2014) showed that the more frequent use of maladaptive ER strategies such as rumination by adolescents with ASD was associated with higher levels of anxiety and depression. Focussing on both internalising and externalising psychopathologies in children and adolescents with ASD, stronger reliance on maladaptive (suppression) and weaker reliance on adaptive (reappraisal) ER strategies has shown to be related to higher levels of negative emotion (Samson et al. 2015a). These increased levels of negative emotion in turn resulted in maladjusted behaviour, such as impulsivity, truancy, and temper tantrums. Thus, although the core features of ASD are not affective in nature, impaired ER may explain why certain psychopathologies often accompany the disorder.

In addition to helping explain comorbid psychopathology, it has been suggested that ER could be useful in explaining the core symptoms of ASD as well (Mazefsky et al. 2013; Richey et al. 2015; Weiss et al. 2014). Core symptoms such as social communicational problems can be understood in light of ER deficits because they require adjustment of internal affective experiences to changing situational demands. Given the problems many individuals with ASD experience in social-affective functioning, it is not surprising that ER might play an important role in characteristics that are central to the disorder. Backing up this supposition, Samson et al. (2014b) have shown that the emotion dysregulation children and adolescents with ASD show compared to TD individuals is related to the severity of several of the core symptoms of ASD: impaired social responsiveness, sensory abnormalities, and especially restricted and repetitive behaviour.

Besides self-report and behavioural research, knowledge of the biological processes involved in ER could help us advance our understanding of ER deficits in e.g. ASD. A small number of studies have indeed used physiological measures to illustrate the relationship between deviant ER patterns and ASD symptomatology. In their review of this literature, Weiss et al. (2014) mention only four studies doing so. One, a study by South et al. (2012), uses skin conductance but focusses more on behavioural inflexibility than actual ER. Three other studies use respiratory sinus arrhythmia (RSA), a measure of parasympathetically mediated heart rate variability. They show that individuals with ASD have lower RSA, which is related to slower emotion recognition (Bal et al. 2010), more social and internalising problems (Neuhaus et al. 2014), fewer social skills, and more problem behaviours (Vaughan Van Hecke et al. 2009). A subsequent RSA study (Guy et al. 2014) reports findings similar to those of Neuhaus et al. (2014): children with ASD were shown to have lower RSA compared to TD children, which was associated with increased anxiety and lower socialisation. While these findings all indicate bad ER, they only provide information about symptoms related to ER, but do not directly measure the construct. An additional point of criticism concerns the interpretation of RSA. While high baseline RSA is suggested to be related to flexible emotional responses, the interpretation of change in RSA depends heavily on context (Mazefsky et al. 2013): effective coping in stressful situations is indicated by an RSA decrease, but in non-stressful situations effective ER is indicated by an RSA increase instead.

In contrast to the RSA studies, two functional magnetic resonance imaging (fMRI) studies did examine ER directly. After instructing children and adolescents to use cognitive reappraisal to modulate their emotional responses to disgusting images, TD individuals showed decreased activity of the insula and amygdala (Pitskel et al. 2014). Individuals with ASD however showed no change in insular activation and had increased activity in the amygdala, even though the affect ratings did not differ between groups. Similar findings were obtained with the use of facial stimuli in an adult population (Richey et al. 2015). Adults with ASD were, compared to TD controls, not able to increase activity in brain regions involved in effortful ER (e.g. striatum, dorsolateral prefrontal cortex) when instructed to think negatively or positively about neutral faces. Together, these neuroimaging studies indicate that the hemodynamic responses that TD individuals show when regulating emotions are not or to a lesser extent observed in individuals with ASD.

The discussed neuroimaging studies succeed in approximating the neurobiological basis of ER deficits in ASD, especially compared to the neurophysiological studies using RSA. However, the technical characteristics of neuroimaging may not be ideal for examining ER. Emotion regulation can be considered a momentary response to stimuli, and its time course can be best assessed by the study of event-related potentials (ERPs). ERPs are calculated using electroencephalography (EEG), which can be sampled in the order of milliseconds. An ERP component that has repeatedly been linked to one specific ER strategy, reappraisal, and that has shown good psychometric properties in studying the concept (Moran et al. 2013), is the late positive potential (LPP).

The LPP starts approximately 400–500 ms after stimulus onset, lasts several 100 ms, and is maximal at the posterior scalp. Hajcak et al. (2006) demonstrated that the LPP is enhanced for affectively arousing (pleasant and unpleasant) compared to neutral pictures. In addition, the potential is reduced when participants are asked to make non-affective compared to affective judgements, indicating that the LPP is not only related to the processing of emotional stimuli but to regulation of these stimuli (ER) as well. This was confirmed by electrophysiological studies focussing on reappraisal. In a study by Hajcak and Nieuwenhuis (2006), adult participants were shown pleasant, unpleasant, and neutral pictures, and were asked just to attend to the picture or to reappraise the content. During passive viewing, the LPP was enhanced for pleasant and unpleasant compared to neutral pictures. Reappraisal of unpleasant stimuli resulted in reduction of the LPP, and the degree of LPP modulation was related to reduction in self-reported emotional intensity. Similar results were obtained for children (Dennis and Hajcak 2009): when asked to provide certain interpretations of unpleasant pictures, the LPP was reduced when giving a neutral compared to a negative interpretation. Stronger modulation was also associated with clinically relevant measures, namely reduced anxious-depressed symptoms, and better parent-reported ER.

Despite these promising results concerning the LPP as a neurobiological marker of reappraisal, and despite the importance of ER in ASD, no research to date has used the LPP as a neurobiological derivative of ER deficits in ASD. The potential has sparsely been used in studying impaired ER in obsessive compulsive disorder (Paul et al. 2016) and schizophrenia (Horan et al. 2013; Strauss et al. 2015), disorders that both show similarities with ASD in symptomatology and aetiology (Couture et al. 2010; Jacob et al. 2009). These studies therefore demonstrate the possibility to examine the LPP not only in people with well-functioning ER but also in clinical populations of which the individuals show ER impairments—at least as determined with the use of self-report and behavioural measures. Because the LPP is sensitive to ER, the LPP change when using ER is possibly lower in individuals high on the autistic spectrum. This would signify an electrophysiological indicator of impaired ER in ASD.

The present article aims to provide an initial examination of the electrophysiological basis of ER deficits in ASD. This examination will be executed building on the autism spectrum hypothesis, which postulates that ASD is not a distinct disorder but a continuum that extends from TD individuals in the general population to diagnosed individuals belonging to the clinical population (Baron-Cohen et al. 2001; Wing 1988). Across this continuum, individuals show different levels of symptom severity. Responses of both non-diagnosed individuals and people belonging to the clinical population offer valuable insights into ASD, since some of the difficulties experienced by diagnosed people are (to a lesser extent) also experienced by TD individuals scoring high on autistic traits (see e.g. De Groot and Van Strien 2016).

The present research comprises two parts: replicating the deviant ER patterns found in self-report and behavioural research on ASD, and extending these findings to electrophysiological measures. With regard to the first part, we will examine whether self-reported ER scores differ between TD individuals scoring low on ASD traits (low-AQs) and TD individuals scoring high (high-AQs), with the group names representing a median split based on the Autism-Spectrum Quotient (AQ) score. Based on the studies by Samson and colleagues, we expect that low-AQs show more adaptive and less maladaptive ER compared to high-AQs. The second set of analyses focusses on the link between ASD and the LPP for one specific ER strategy: reappraisal. The main question is whether low-AQs and high-AQs differ in LPP-amplitude change when instructed to use reappraisal compared to passive viewing. Based on Hajcak and Nieuwenhuis (2006), and on the self-report and behavioural studies showing ER deficits in individuals with ASD, we expect that high-AQs show a less pronounced LPP decrease when asked to down-regulate elicited negative feelings (decrease effect), and that they show a less pronounced LPP increase when asked to up-regulate elicited positive feelings (increase effect), compared to low-AQs. Besides the influence of reappraisal, we will also examine the possible differences between low and high-AQs in their initial LPP response to processing arousing (pleasant and unpleasant) stimuli compared to neutral stimuli. This latter analysis focusses on emotional reactivity rather than on ER, but was included since most examinations of the LPP look at both emotional reactivity and ER (Hajcak and Nieuwenhuis 2006; Hajcak et al. 2006; Paul et al. 2016). However, hypothesising on the outcome of emotional reactivity is difficult. A self-report study found increased emotional reactivity in individuals scoring high on the spectrum (Pisula et al. 2015). A behavioural study found equally strong reactivity (Samson et al. 2014a). Contrary to both, an fMRI study showed reduced responses to happy versus neutral faces in individuals with ASD compared to TD controls, with unaffected siblings demonstrating intermediate levels of reactivity (Spencer et al. 2011). Focussing on the nature of the stimulus presentation, a study using pupillometry showed reduced reactivity to backward-masked but equal reactivity to consciously presented stimuli in ASD (Nuske et al. 2014). Finally, a review on emotion impairments in ASD states that the conflicting findings on emotional reactivity may be the result of the impairment being domain-specific: whereas the ‘hardware’ for emotional reactivity seems to be functional, individuals with ASD may show abnormal reactivity only for social stimuli (Nuske et al. 2013). Because of the lack of univocal findings on reactivity, no hypotheses were formulated for this construct.

## Methods

### Participants

Participants were 60 TD students from the Institute of Psychology who participated in reward for course credit. The sample consisted of 19 male and 41 female participants with a mean age of *M* = 20.25 (*SD* = 1.85), range 18–26 years. Participants were informed about the nature of the measurements (EEG) beforehand, and informed consent was obtained from all participants included in the study. All procedures performed were in accordance with the ethics standards of the institutional review board and with the 1964 Helsinki declaration and its later amendments.

### Materials and Stimuli

#### Autism-Spectrum Quotient

The Autism-Spectrum Quotient (AQ, Baron-Cohen et al. 2001) is a continuous and quantitative self-report measure of autistic traits in adults of normal intelligence. The questionnaire consists of 50 questions, divided into five subscales of ten items each: social skill, attention switching, attention to detail, communication, and imagination. Items are answered on a 4-point Likert-scale: definitely agree, slightly agree, slightly disagree, definitely disagree. Completing all items takes approximately 15 min. Both the original English version of the test and its Dutch translation show satisfactory psychometric properties (Baron-Cohen et al. 2001; Hoekstra et al. 2008). The binary scoring scheme as originally proposed by Baron-Cohen et al. (2001) ignores the degree of agreement or disagreement. In line with Hoekstra et al. (2008), we used a 4-point rating scale, which has been shown to improve the reliable range of measurement significantly (Murray et al. 2016). This resulted in a minimum total score of 50 (the individual reports having no autistic traits) and a maximum score of 200 (the individual reports having the full range of autistic traits). As could be expected based on score variability, reliability was better when using the full-range scoring scheme. Cronbach’s alfa was α = .82 for the composite score (as opposed to α = .71 using binary scores), α = .75 (α = .55) for social skill, α = .73 (α = .65) for attention switching, α = .63 (α = .53) for attention to detail, α = .62 (α = .54) for communication, and α = .46 (α = .28) for imagination. These reliabilities were similar to those found by Hoekstra et al. (2008).

#### Questionnaire of Emotion Regulation for Adults

The Questionnaire of Emotion Regulation for Adults (FEEL-E, Grob and Horowitz 2014) is a self-report questionnaire in which participants indicate in approximately 15 min what they do or think when being angry (24 items), scared (24 items) or sad (24 items). The instrument differentiates between six adaptive (problem-oriented action, acceptance, cognitive problem-solving, reappraisal, evoking positive feelings, forgetting) and six maladaptive (withdrawal, self-blame, resignation, rumination, negative thinking, other-blame) ER strategies. Every item is answered and scored on a 5-point scale: almost never, seldom, sometimes, often, almost always. Scores can be calculated for both adaptive and maladaptive strategies and for each emotion separately. The psychometric properties of the Dutch adaptation of the scales were satisfactory, and examination of the criterion validity showed that people with ASD scored lower on adaptive strategies than both the normative group and individuals with other psychiatric disorders (Punt 2015). The data from the present study yielded a Cronbach’s alfa of α = .92 for the adaptive scale, and α = .88 for the maladaptive scale.

#### EEG Stimuli

The stimulus set consisted of 120 International Affective Picture System (IAPS) pictures and was identical to the set used by Hajcak and Nieuwenhuis (2006): 40 pleasant pictures, 40 unpleasant pictures, and 40 neutral pictures.^1^ Because Hajcak and Nieuwenhuis (2006) forgot to report one neutral item, an extra item was added. The reported neutral items consisted of people (5 items), objects (25 items), and nature (9 items). We added one nature item (5120, ‘pine needles’), which changed the mean neutral valence and arousal rating with 0.01. Normative valence ratings significantly differed between all picture categories, with unpleasant pictures scoring lowest (*M* = 2.52), neutral pictures scoring intermediate (*M* = 5.04), and pleasant pictures scoring highest (*M* = 7.01), all *p*-values <.001. Normative arousal ratings also significantly differed between all picture categories: neutral pictures had the lowest arousal ratings (*M* = 2.75), and differed significantly from pleasant and unpleasant pictures, both *p*-values <.001. The difference between pleasant (*M* = 5.49) and unpleasant (*M* = 6.03) pictures was small but significant (*p* = .036). Multiple comparisons were Games-Howell corrected.

### Procedure

The measures were part of a larger study examining both self-report and brain correlates of impairments often seen in ASD. The total experimental session took approximately 90 min, including breaks. First, participants filled out three questionnaires, including the AQ and the FEEL-E. Thereafter, the participant was seated in a comfortable chair in a light and sound-attenuated EEG room. After a brief description of the experimental task, the electrodes were placed. Then detailed task instructions were given. Participants were presented with both a passive viewing block and an ER block. The total viewing time was approximately 15 min, including a short break between the view and the ER block. The order in which the blocks were presented, was counterbalanced.

The passive viewing block consisted of all 120 pictures (neutral, pleasant, unpleasant). The instruction screen told the participant to only pay attention to the stimuli. Then, all 120 pictures were presented in random order. Every picture was presented for 1000 ms, and was preceded by a blank interval for 1400–1600 ms, and a fixation word (‘view’ in Dutch) for 1000 ms to remind the participant of the task.

The ER reappraisal block consisted of the 40 pleasant and the 40 unpleasant pictures. Stimuli were again randomly presented for 1000 ms, and preceded by a variable interval and a fixation word. In line with previous studies linking ER to the LPP, the ER instructions given to the participants were focussed on reappraisal. Reappraisal was explained as reinterpreting a picture in such a way that you feel differently about it. Two possible situations were illustrated: when seeing an unpleasant picture, you can reinterpret it so that it no longer elicits a negative feeling, or when seeing a pleasant picture, you can reinterpret it so that the positive feeling you have is increased. Examples were given as well. Down-regulation of negative emotions was explained with the example of a funeral: seeing a picture of this makes you feel sad, but you can decrease that sad feeling by imagining that the deceased was very old and lived a beautiful life, or that the shown event was staged. Up-regulation of positive emotions was explained with the example of a graduation: seeing a picture of this makes you feel good, and you can increase that good feeling by imagining that it was your own graduation or that it was followed by a nice party. Only up-regulation of pleasant pictures and down-regulation of unpleasant pictures was used, since the treatment-relevant outcomes of ER are usually making good feelings even better and turning bad feelings around. The fixation word used in the ER block was the Dutch word for ‘nicer’, meant to remind participants to interpret the situation in such a way that the resulting feelings improved compared to the initial feelings. The appropriateness of the reminder word ‘nicer’ was supported by the fact that several participants interrupted the ER instruction by stating that ‘they had to make everything nicer’. The instruction phase ended with asking the participants whether they had completely understood the instructions and whether they had any questions pertaining to the task.

### Electrophysiological Recordings and Signal Processing

EEG was recorded using a 32-channel amplifier and ActiveTwo data acquisition software (Biosemi, Amsterdam, The Netherlands). Ag/AgCl active electrodes were placed on the scalp by means of a head cap according to the 10–20 placing system. The electro-oculogram (EOG) was recorded by placing flat electrodes above and below the left eye (vertical EOG) and at the outer canthi of both eyes (horizontal EOG). Referencing was done via two electrodes placed on the mastoids. An active (CMS—common mode sense) and a passive (DRL—driven right leg) electrode were used to comprise a feedback loop for amplifier referencing. All signals were digitised with a sampling rate of 512 Hz.

The data were analysed offline with BrainVision Analyzer 2 (Brain Products, Gilching, Germany). All EEG channels were referenced to the mathematically linked mastoid electrodes. A low cut-off of 0.1 Hz and a high cut-off of 30 Hz were applied, together with a notch filter of 50 Hz to filter out artefact caused by electrical power lines. Data were segmented into epochs from 100 ms pre-stimulus onset till 1000 ms post-stimulus onset. Ocular artefact corrections were done using the Gratton and Coles algorithm (Gratton et al. 1983). After this, baseline correction was applied over the selected 100 ms pre-stimulus onset period. Automatic artefact rejection allowed a minimal amplitude of −100 µV and a maximal amplitude of 100 µV. Epochs were classified according to picture type and instruction, yielding five conditions (view-pleasant, view-unpleasant, view-neutral, ER-pleasant, ER-unpleasant). Data of participants with less than 30 (out of 40) segments in at least one condition was scrutinised to identify the electrodes responsible for this low number. Analysis-relevant electrodes for which more than 5% of data was removed, were interpolated by spherical spline. This resulted in an average number of *M* = 38.90 (*SD* = 2.01) valid segments used for averaging across participants, which did not differ between conditions, *F*(4, 295) = 0.20, *p* = .939, $\upeta _{\text{p}}^2$ < 0.01.

**Fig. 1:**
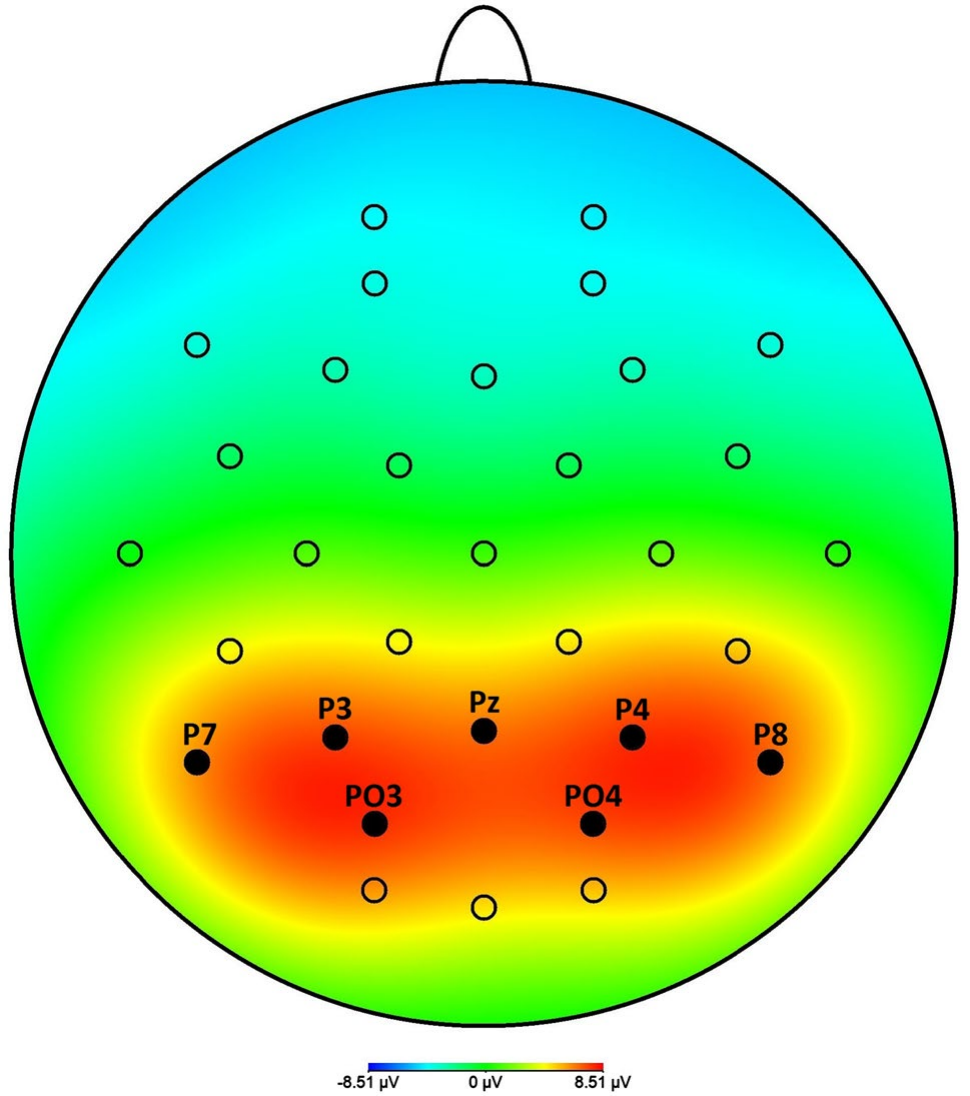
Head view image of averaged activity in the 400–800 ms time window across all conditions (view-pleasant, view-unpleasant, view-neutral, ER-pleasant, ER-unpleasant) and all participants (*N* = 60)

The time epoch and electrode cluster used for pooling oscillatory activity were based upon both previous findings and visual inspection of the data. The LPP has been shown to be maximal at posterior sites, and a head view image averaging all conditions indeed showed that for the present data, activity was strongest at the Pz, P3, P4, P7, P8, PO3, and PO4 electrodes (see Fig. 1). Therefore, the amplitudes of these electrodes were averaged. A time epoch of 400–800 ms was used across all analyses.

### Analyses

First, a set of preliminary analyses consisting of *t*-tests examined the characteristics of the dependent variable, AQ score. Then, independent samples *t*-tests were used to determine whether low-AQs differed from high-AQs in their use of adaptive and maladaptive ER strategies. The low-AQ and high-AQ group were based on a median split (median = 95), with tied scores assigned to the low-AQ group. To provide a more comprehensive view of the relationship between autistic traits and ER, a correlation analysis was performed in which the Pearson’s correlations between all AQ sub-scores and all FEEL-E sub-scores were calculated.

With regard to the electrophysiological data, group differences in passive viewing (emotional reactivity) and reappraisal (emotion regulation) were examined. First, we examined the possible difference between low-AQs and high-AQs in LPP-change in response to processing arousing (pleasant and unpleasant) stimuli compared to neutral stimuli. This was done with the use of a mixed two-way ANOVA: 2 (group, between: low-AQ, high-AQ) × 3 (picture type, within: view-neutral, view-pleasant, view-unpleasant). Second, we examined the possible difference between low-AQs and high-AQs in LPP-change when participants were instructed to use reappraisal compared to passive viewing. To this end, the mean amplitudes were subjected to a mixed three-way ANOVA: 2 (group, between: low-AQ, high-AQ) × 2 (valence, within: pleasant, unpleasant) × 2 (action, within: passive viewing, reappraisal). In addition, we examined whether the counterbalancing order impacted the results. To extend the localization properties of the 2D solution to a 3D solution, an explorative low-resolution electromagnetic tomography (LORETA) algorithm was performed to examine which areas were most activated during the tasks.

Across all analyses, an alpha level of α = .05 was used. In case of multiple comparisons, alpha was corrected using the Sidak procedure. If possible, effect sizes were reported as $\upeta _{\text{p}}^2$ for easy comparison.

## Results

### The Autism-Spectrum Quotient

The AQ scores ranged from 65 to 127 (low-AQ group range 65–95, high-AQ group range 96–127). The distribution was approximately normal as determined by visual inspection of the normal Q–Q-plot. Table 1 shows the mean AQ scores and standard deviations per sex and group. The mean AQ score was comparable to findings from other social sciences students samples (Hoekstra et al. 2008). The median-split based low-AQ group and the high-AQ group did not significantly differ in age, *t*(58) = 1.16, *p* = .250, 95% CI [−1.51, 0.40], $\upeta _{\text{p}}^2$ = 0.02. The difference in AQ score between men and women was borderline significant, *t*(58) = 1.86, *p* = .068, 95% CI [−0.46, 12.82], $\upeta _{\text{p}}^2$ = 0.06, with men having higher scores than women. None of the participants scored above the clinical cut-off score of 32 (Baron-Cohen et al. 2001) as determined by the binary scoring scheme (present range is 3–26 out of 0–50).

**Table 1:** Mean (SD) AQ scores and ranges per gender and group

	Low-AQ	High-AQ	Total
Men	88.00 (7.77) *n* = 7	106.58 (8.51) *n* = 12	99.74 (12.22) *n* = 19
Women	87.19 (8.49) *n* = 26	104.60 (8.09) *n* = 15	93.56 (11.83) *n* = 41
Total	87.36 (8.23) *n* = 33	105.48 (8.18) *n* = 27	95.52 (12.20) *n* = 60

### Self-Report Analyses

#### Do Low-AQs and High-AQs Differ in Terms of Their Use of Adaptive ER?

**Fig. 2:**
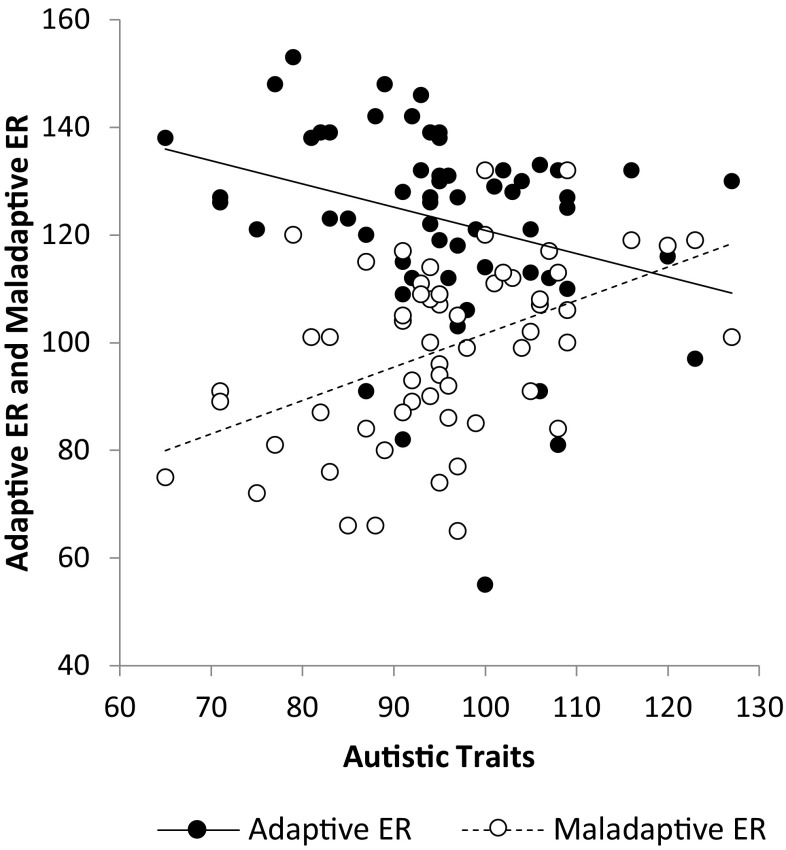
The relationship between autistic traits (*x*-axis) and the use of adaptive and maladaptive ER strategies (*y*-axis)

The adaptive ER scores ranged from 55 to 153, with a mean score of *M* = 122.82 (*SD* = 17.75). This mean value was comparable to the mean score for a TD population found in previous psychometric evaluation of the questionnaire: *M* = 123.40 (Punt 2015). The total adaptive ER scores violated both the normality assumption (negative skew: *D*(60) = 0.13, *p* = .019) and the homoscedasticity assumption, and therefore simple bootstrapping (1000 samples) was used to calculate corrected confidence intervals. An independent samples *t-*test showed that TD individuals scoring high on the autistic spectrum (*M* = 115.78, *SD* = 18.23) made significantly less use of adaptive ER strategies than TD individuals scoring low on the autistic spectrum (*M* = 128.58, *SD* = 15.32), *t*(58) = 2.96, *p* = .005, 95% BCa CI [4.68, 20.74], $\upeta _{\text{p}}^2$ = 0.13. The relationship between AQ score and the use of adaptive ER is shown in Fig. 2 (solid line).

#### Do Low-AQs and High-AQs Differ in Terms of Their Use of Maladaptive ER?

The maladaptive ER scores ranged from 65 to 132, *M* = 98.88 (*SD* = 16.24). This mean value was comparable to the mean score for a TD population found in previous psychometric evaluation of the questionnaire: *M* = 98.40 (Punt 2015). The distribution was approximately normal as determined by visual inspection of the normal Q–Q-plot. An independent samples *t-*test showed that TD individuals scoring high on the autistic spectrum (*M* = 104.19, *SD* = 16.02) made significantly more use of maladaptive ER strategies than TD individuals scoring low on the autistic spectrum (*M* = 94.55, *SD* = 15.31), *t*(58) = 2.38, *p* = .021, 95% CI [−17.76, −1.52], $\upeta _{\text{p}}^2$ = 0.09. The relationship between AQ score and the use of maladaptive ER is also shown in Fig. 2 (dashed line).

#### How are Autistic Traits Related to Adaptive ER Strategies?

Table 2 shows the correlations between the AQ scores and the use of adaptive ER strategies. The FEEL-E sub-scores are split up into the use of adaptive ER when experiencing three separate emotions (covering all strategies) and into the use of six separate strategies (covering all emotions). Most correlations were negative and small (*r* = 0.10) to medium (*r* = 0.30). The total AQ score was significantly related to the total FEEL-E adaptive score: having more autistic traits was related to less use of adaptive ER strategies. Two AQ sub-scores were not related to the use of adaptive ER: imagination and attention to detail. The other AQ sub-scores were almost primarily related to the use of adaptive ER when feeling angry, and not when feeling scared or sad: the more social, attention switching and communicational autistic traits individuals exhibited, the less they used adaptive ER strategies when feeling angry. With respect to the separate ER strategies, only evoking positive feelings was significantly related to the total AQ score, though problem-oriented action, acceptance, and reappraisal were significantly related to one or more AQ sub-scores. The use of cognitive problem-solving and forgetting were not significantly related to any AQ score.

**Table 2:** Correlations between AQ sub-scores and adaptive FEEL-E scores for three separate emotions and six adaptive ER strategies

	AQ social skills	AQ attention switching	AQ communication	AQ imagination	AQ attention to detail	AQ total
FEEL-E emotions						
Angry	−.35**	−.37**	−.41**	.01	.03	−.35**
Scared	−.21	−.02	−.11	−.12	−.03	−.15
Sad	−.24	−.25	−.26*	−.05	.03	−.25
FEEL-E ER strategies						
Problem-oriented action	−.34**	−.15	−.25	−.05	−.01	−.25
Acceptance	−.37**	−.28*	−.26*	.13	.07	−.24
Cognitive problem-solving	−.22	−.08	−.15	−.03	.05	−.14
Reappraisal	−.03	−.28*	−.18	−.05	.06	−.15
Evoking positive feelings	−.28*	−.20	−.33**	−.19	−.07	−.33*
Forgetting	−.20	−.22	−.24	−.06	−.04	−.24
FEEL-E adaptive total	−.32*	−.27*	−.31*	−.06	.01	−.30*

*Is significant at .05 level (2-tailed)

**Is significant at .01 level (2-tailed)

#### How are Autistic Traits Related to Maladaptive ER Strategies?

Table 3 shows the correlations between the AQ scores and the use of maladaptive ER strategies. The FEEL-E sub-scores are split up into the use of maladaptive ER when experiencing three separate emotions (covering all strategies) and into the use of six separate strategies (covering all emotions). Most correlations were positive and medium (*r* = 0.30) to large (*r* = 0.50). The total AQ score was significantly related to the total FEEL-E maladaptive score, meaning that having more autistic traits was accompanied by using more maladaptive ER strategies. The AQ imagination and attention to detail sub-scales were not related to the use of maladaptive ER, just as they were not related to the use of adaptive ER. However, the other AQ sub-scores and the total AQ score were all significantly related to the use of maladaptive ER when feeling angry, scared, and sad: individuals who exhibited more autistic traits used more maladaptive ER strategies when confronted with all negative emotions. The separate maladaptive ER strategies were also linked more strongly to AQ scores compared to the link between AQ and adaptive ER; other-blame was the only maladaptive ER strategy that was not significantly linked to any of the AQ scores. Especially the use of withdrawal, self-blame, and resignation was strongly related to having more autistic traits.

**Table 3:** Correlations between AQ sub-scores and maladaptive FEEL-E scores for three separate emotions and six maladaptive ER strategies

	AQ social skills	AQ attention switching	AQ communication	AQ imagination	AQ attention to detail	AQ total
FEEL-E emotions						
Angry	.47**	.53**	.52**	.09	−.08	.48**
Scared	.36**	.31*	.28*	.10	.06	.35**
Sad	.38**	.37**	.38**	−.01	.10	.39**
FEEL-E ER strategies						
Withdrawal	.53**	.36**	.35**	.15	.20	.50**
Self-blame	.27*	.39**	.35**	.11	−.16	.32*
Resignation	.42**	.38**	.39**	−.05	−.02	.36**
Ruminating	.22	.26*	.08	−.20	<.01	.14
Negative thinking	.27*	.23	.35**	.28*	.11	.37**
Other-blame	−.08	.03	.10	−.07	−.08	−.03
FEEL-E maladaptive total	.46**	.46**	.45**	.07	.03	.47**

*Is significant at .05 level (2-tailed)

**Is significant at .01 level (2-tailed)

### Electrophysiological Analyses

**Fig. 3:**
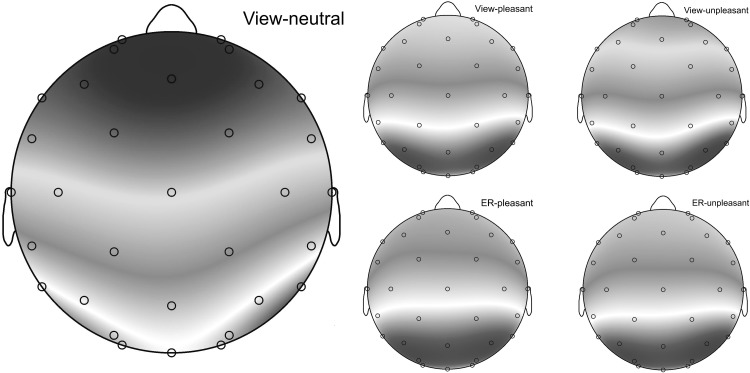
Averaged topography (400–800 ms) in response to the view-neutral condition and the experimental conditions: view-pleasant, view-unpleasant, ER-pleasant, ER-unpleasant

**Fig. 4:**
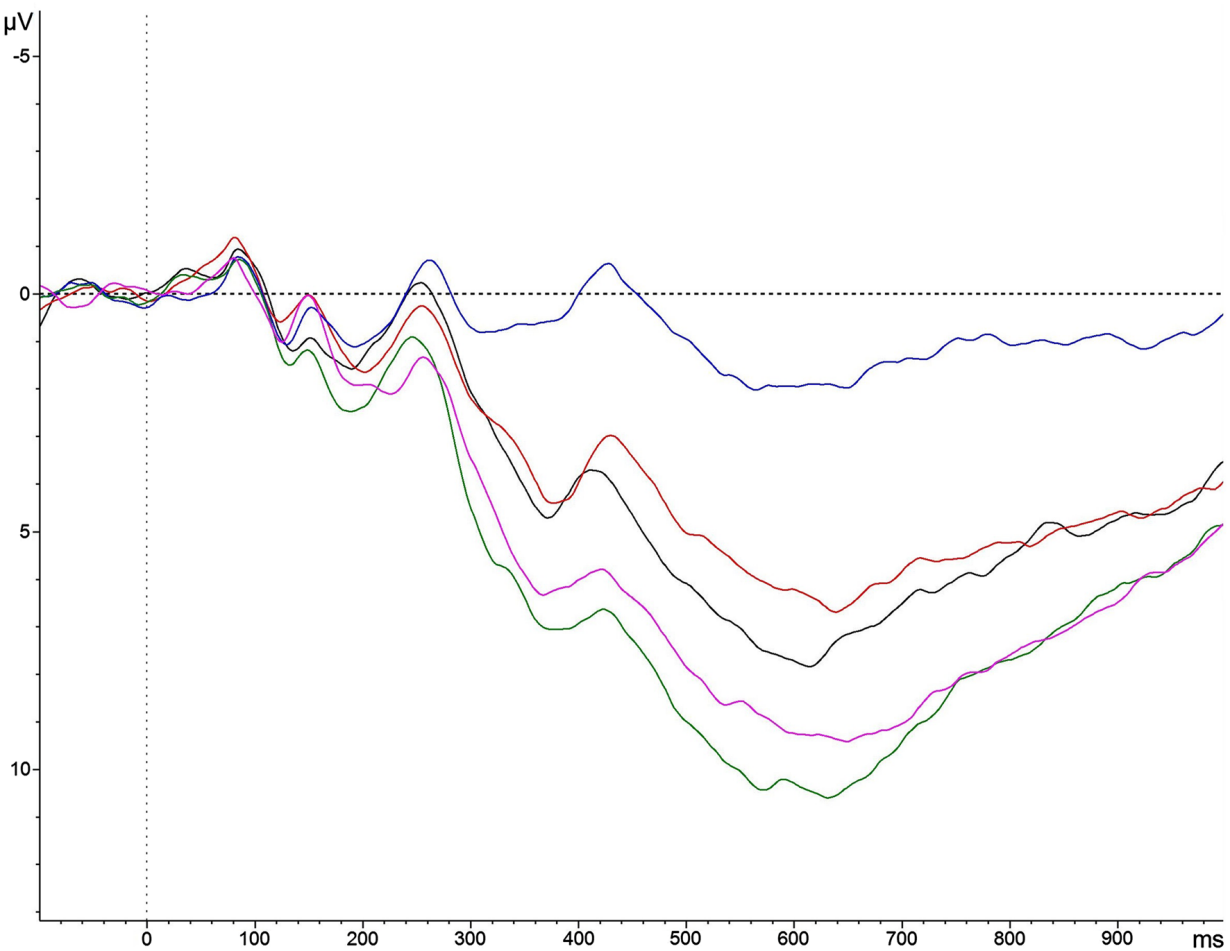
The ERP waveforms at the Pz electrode for the view-neutral condition (*blue*), the view-pleasant condition (*black*), the view-unpleasant condition (*red*), the ER-pleasant condition (*green*), and the ER-unpleasant condition (*purple*). (Color figure online)

Table 4 shows the mean ERP waveform values per condition and group. Figure 3 shows how the effects of the different conditions are distributed across the scalp, showing a clear posterior pattern for all experimental conditions. The ERP waveforms at the Pz electrode are shown in Fig. 4.

**Table 4:** Mean (SD) LPP 400–800 ms area measures (in µV) for the parietal cluster per condition and group

	Low-AQ	High-AQ	Total
View pleasant	7.26 (3.25)	7.38 (3.58)	7.32 (3.38)
View unpleasant	6.60 (3.48)	7.58 (3.67)	7.04 (3.57)
View neutral	4.14 (3.13)	3.93 (2.94)	4.04 (3.02)
ER pleasant	8.78 (3.42)	9.78 (3.58)	9.23 (3.50)
ER unpleasant	8.88 (3.58)	8.95 (4.02)	8.91 (3.75)
Total	7.13 (2.77)	7.53 (2.99)	7.31 (2.85)

#### Emotional Reactivity

The 2 × 3 RM ANOVA showed a significant main effect of condition, *F*(2, 116) = 53.17, *p* < .001, $\upeta _{\text{p}}^2$ = 0.48. Pairwise comparisons indicated that viewing neutral images elicited significantly lower amplitudes than viewing both pleasant (*p* < .001, 95% CI [2.40, 4.18]) and unpleasant (*p* < .001, 95% CI [2.16, 3.95]) images. The view-pleasant and view-unpleasant conditions did not differ significantly from each other, *p* = .878, 95% CI [−0.61, 1.07]. The effect of group was not significant, *F*(1, 58) = 0.15, *p* = .697, $\upeta _{\text{p}}^2$ < 0.01. Neither was the interaction between group and condition, *F*(2, 116) = 1.48, *p* = .231, $\upeta _{\text{p}}^2$ = 0.02.

#### Emotion Regulation

The 2 × 2 × 2 RM ANOVA showed a significant main effect of action, *F*(1, 58) = 32.66, *p* < .001, $\upeta _{\text{p}}^2$ = 0.36, with higher amplitudes for reappraisal (9.10 µV) compared to passive viewing (7.21 µV). None of the other main effects or interaction effects reached significance, meaning that there was no difference across valences [*F*(1, 58) = 1.25, *p* = .268, $\upeta _{\text{p}}^2$ = 0.02], groups [*F*(1, 58) = 0.48, *p* = .493, $\upeta _{\text{p}}^2$ < 0.01], or any of the interactions between action, group, and valence (all *p*-values > .05).

To examine the impact of the counterbalancing, we executed a second RM ANOVA with counterbalancing condition included as well. The main effect of counterbalancing condition was not significant [*F*(1, 56) = 0.54, *p* = .464, $\upeta _{\text{p}}^2$ = 0.01]. Two interactions reached significance: the interaction between action and counterbalancing [*F*(1, 56) = 10.72, *p* = .002, $\upeta _{\text{p}}^2$ = 0.16] and the higher-order interaction between action, valence, and counterbalancing [*F*(1, 56) = 7.73, *p* = .007, $\upeta _{\text{p}}^2$ = 0.11]. Amplitudes were higher in the ER compared to the view condition, but if participants first performed the ER condition, the difference between conditions disappeared for the unpleasant stimuli.

#### Tomography

**Fig. 5:**
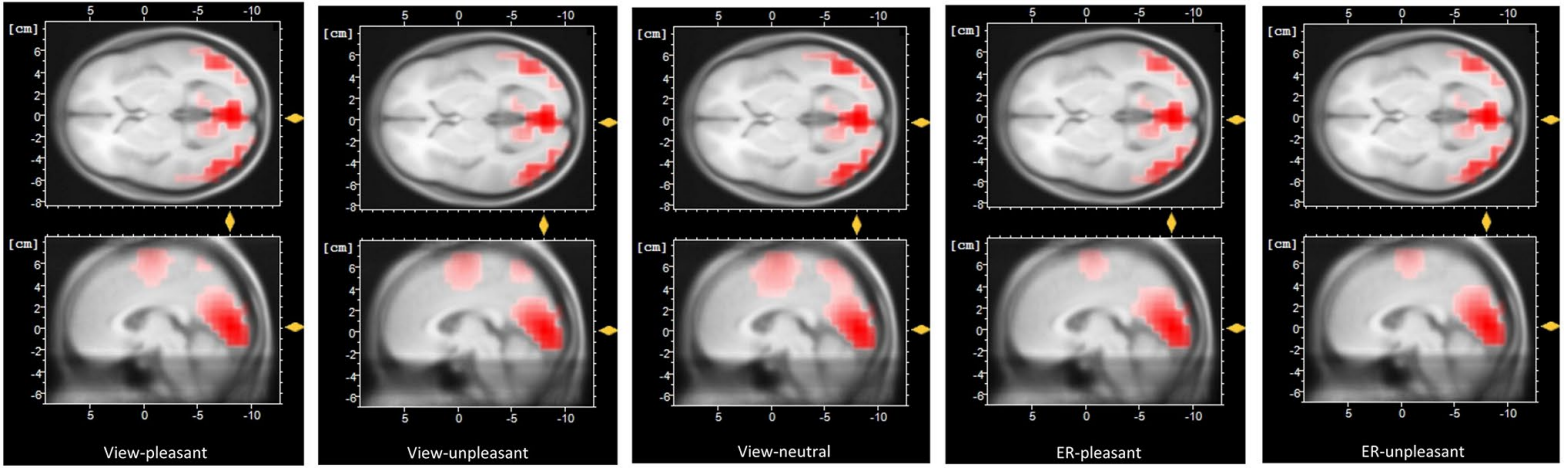
Averaged tomography (400–800 ms) in response to the view-pleasant, view-unpleasant, view-neutral, ER-pleasant, and ER-unpleasant condition

The result of the LORETA algorithm conversion is shown in Fig. 5. Averaged across all conditions, the lingual gyrus was the most active site since the best match was found at 2 mm in the lingual gyrus (*x* = −3, *y* = −81, *z* = 1). The precuneus area was activated during the view conditions, but not during reappraisal.

## Discussion

The present study employed both self-report and brain measures to examine the capacity to regulate emotions in individuals scoring low or high on the autistic trait continuum. With regard to the self-report measures, it was examined whether individuals scoring high on autistic traits show a deviant ER pattern. In line with studies on individuals from the clinical population (Samson et al. 2012, 2014a, b, 2015a, b), people scoring high on autistic traits reported using fewer adaptive and more maladaptive ER strategies than individuals scoring low on autistic traits. Our results thus demonstrate that similar associations between autistic traits and ER strategies can be found in both a clinical and a non-clinical sample, which is consistent with the broad autism spectrum approach.

It should be noted that our results were partly discordant from one study that showed that, although individuals with ASD make more use of maladaptive ER strategies, they do not differ in the amount of adaptive ER strategies that they employ (Mazefsky et al. 2014). This discrepancy can be explained by the fact that Mazefsky et al. (2014) employed the Response to Stress Questionnaire (RSQ) to assess ER. The RSQ does for the most part focus on sadness and fear, and not on anger. Our self-report results (see Table 2) indicate that the amount of autistic traits is negatively associated with ER strategies in response to feeling angry rather than feeling scared or sad. Therefore, it is not surprising that the RSQ as employed by Mazefsky et al. (2014), which mainly focusses on feelings other than anger, does not yield a difference in the use of adaptive ER strategies between individuals with and without ASD.

Many studies fail to look at a wider palate of ER strategies, often exclusively focussing on the use of reappraisal and suppression (e.g. Samson et al. 2012, 2015a). The present self-report data show that this restricted focus is not necessarily justifiable, since our findings indicate that reappraisal is not the adaptive ER strategy most strongly related to autistic traits: it only correlated significantly with the attention switching sub-score, and it did not correlate significantly with total AQ. Both acceptance and evoking positive feelings were correlated more strongly with autistic traits than reappraisal was. Although we cannot comment on the link between suppression and autistic traits, we found that several other maladaptive ER strategies (withdrawal, self-blame, resignation, negative thinking) were also linked to autistic traits. Besides emphasising the need to examine a less limited range of ER strategies, these findings are relevant for clinical practice as well. Knowing that individuals with more autistic traits make less use of acceptance and evoke less positive feelings when dealing with a difficult emotional situation, and that they make more use of withdrawal, self-blame, resignation, and negative thinking, certainly has implications for therapy development.

With regard to the electrophysiological findings, the ERP analyses examined the possible difference between low-AQs and high-AQs in LPP-change in response to viewing arousing (pleasant and unpleasant) stimuli compared to neutral stimuli (emotional reactivity), and in response to reappraising arousing stimuli compared to passively viewing those stimuli (emotion regulation). The emotional reactivity analyses showed that participants had a stronger electrophysiological response to images with emotional content compared to neutral images, as indicated by a larger LPP amplitude. However, this effect did not differ between individuals scoring low and high on autistic traits. These findings fit with a behavioural study indicating equally strong reactivity in individuals with ASD compared to TD individuals (Samson et al. 2014a). However, it goes against several other studies showing a difference in emotional reactivity between those who score low and those who score high on autistic traits (Nuske et al. 2014; Pisula et al. 2015; Spencer et al. 2011).

With regard to the ERP analyses focussing on ER, the findings did not replicate previous research. In contrast to Hajcak and Nieuwenhuis (2006), the LPP was not attenuated when participants were asked to use reappraisal. In fact, the electrophysiological response even increased. This was to be expected for reappraising pleasant images for the better, but was the opposite of what previous studies found when reappraising unpleasant images. With regard to the unpleasant images, we found an effect of counterbalancing condition: amplitudes were generally higher in the ER compared to the view condition, but if participants first performed the ER condition, the difference between conditions disappeared for the unpleasant stimuli. Finally, the electrophysiological data did not show a difference between individuals scoring low versus high on the autistic spectrum. This could be because reappraisal was not strongly related to autistic traits, at least according to the self-report data. Therefore, future studies on the electrophysiological basis of ER might benefit from looking at other ER strategies as well. Another possibility is that the present sample consisting of only TD individuals did not show enough variability in autistic traits. However, the use of several self-reported ER strategies was successfully correlated with AQ score, indicating that the variability in autistic traits across the sample was at least large enough to show an effect on self-reported ER.

One possible explanation for the unexpected increase instead of decrease in response to reappraising unpleasant stimuli (when the participant did the passive viewing condition first) is that the experimental manipulation failed, meaning that participants did not successfully lower their emotional response to the unpleasant stimuli as instructed. This possibility remains open since we did not ask the participants whether they thought they had been successful in applying the reappraisal strategy. So, future studies should check whether participants implemented ER as instructed, and whether participants implement the strategy automatically when they are not instructed to do so (e.g. in the passive viewing condition). However, for the present case, both situations are not likely to have led to the observed increased amplitude when reappraising unpleasant images versus passively looking at them. After all, if participants fail in reappraising unpleasant stimuli, the amplitude would be equal to the amplitude observed when passively looking at the stimuli, and not increased. Likewise, if people automatically reappraise stimuli when they are instructed to passively look at them, the expected outcome would be an equal amplitude across the passive viewing and the reappraisal condition, since reappraisal is then applied in both conditions (which might have been the case for unpleasant images in participants who first performed the ER condition). Hence, the increased amplitude when reappraising unpleasant stimuli compared to passively looking at them is not what could be expected if the experimental manipulation had failed. Anyway, the unexpected larger LPP amplitudes that we have found in this condition might indicate that the instruction to reappraise unpleasant images induced more arousal in the participants, especially when this condition followed the view condition.

An alternative explanation for the unexpected increase instead of decrease in response to reappraising unpleasant stimuli is the chosen design. The present reappraisal instructions focused on improving the emotional outcome of the presented stimuli, hence the reminder word ‘nicer’. However, asking participants to reappraise unpleasant stimuli by changing the meaning of the stimulus does not necessarily mean that the emotion and thereby the LPP attenuates. Reappraising a picture of a gun could e.g. take place by not looking at it as a dangerous weapon but by thinking of it as an action movie you are about to see. This changes the valence from unpleasant to pleasant, but does not necessarily change the arousal, and therefore does not change the LPP. Because we are asking for change and not attenuation of the experienced emotions, the LPP does not decrease after reappraisal of unpleasant images, and can even increase during this process.

In addition to explaining the present failure to attenuate the electrophysiological response to unpleasant stimuli with the use of reappraisal, the valence-changing account also explains previous reappraisal studies that were successful in modulating the LPP. This is e.g. the case for studies explicitly instructing participants to decrease or increase elicited emotions (Hajcak and Nieuwenhuis 2006; Krompinger et al. 2008; Moser et al. 2006; Schönfelder et al. 2014), and for studies inducing a non-affective instead of an affective context (Dennis and Hajcak 2009; Hajcak et al. 2006). Another example is formed by a study using a more ecologically valid design, showing that LPP amplitudes of unpleasant images are attenuated when participants think the images were depicting art as compared to real scenes (Van Dongen et al. 2016). However, using a non-valence-changing paradigm does not guarantee successful LPP modulation. Even though Langeslag and Van Strien (2010) explicitly instructed participants to either increase or decrease the elicited emotions, only the increase-instructions impacted the LPP. Another study using a non-valence-changing paradigm found increased LPP amplitudes in both the enhance and decrease condition, similar to the present findings (Wu et al. 2013).

The issue of the operationalisation of reappraisal (in our case: changing the valence) represents a fundamental problem: what does reappraisal mean? Despite all discussed LPP studies claiming to use the reappraisal construct, they do not convey a univocal interpretation of the concept. The prevailing opinion is that reappraisal encompasses a decrease or increase of emotional intensity, though this is in fact the end goal of ER strategies in general. Operationalising reappraisal as changing the way one thinks about emotional stimuli and thus turning around the accompanying feelings does differentiate reappraisal from other ER strategies. In addition, this interpretation seems to fit with what participants think, since several of them interrupted the present reappraisal instructions by stating that ‘they had to make everything nicer’. However, this interpretation was presently shown to be unfit to be examined with the use of the LPP, since successfully changing the valence does not necessarily result in LPP modulation. The conflicting findings across studies using different operationalisations of reappraisal should be addressed in future studies. In short, the field requires a more detailed examination of the meaning and electrophysiological consequences of different interpretations of the reappraisal construct.

Lastly, the localization properties of the 2D solution were extended to a 3D solution using a LORETA algorithm. These tomography findings show that the most active site across all conditions was the lingual gyrus, which is sensible considering its role in processing emotional stimuli (Goldin et al. 2008). However, it does raise the question why the lingual gyrus was also the most active posterior site during the view neutral condition. The only differentiation found across conditions was in the precuneus, which was activated during view conditions, but not during reappraisal. A possible explanation for this pattern is a change in the default mode network between viewing images and engaging in goal-directed actions when reappraising, which is related to decreases in tonic activity (Cavanna and Trimble 2006).

To conclude, the present findings show that studies focused on ER in both behaviour and brain should expand the range of ER strategies they focus on, and should operationalise the examined ER strategies properly. In addition, studies on ER should focus more on pathological populations for which impaired ER is not a diagnostic criterion, but for which ER difficulties are often observed nonetheless. From the present findings, it is clear that individuals high on the autistic spectrum show impaired ER. However, one should keep in mind that the present findings apply to a TD population. A logical next step would be to extend these findings to a diagnosed population as well to determine exactly which ER strategies are impaired and how this shows in the brain. Knowing this could prevent both research and clinical practice from putting time and effort into the wrong domains.

## Footnotes

1 Numbers of the IAPS pictures used. *Pleasant* 1601, 2000, 2070, 2080, 2091, 2092, 2165, 2311, 2340, 4002, 4180, 4220, 4290, 4532, 4572, 4608, 4658, 4659, 4660, 4664, 4800, 4810, 5470, 5621, 5626, 5628, 7325, 8021, 8032, 8080, 8200, 8210, 8280, 8320, 8330, 8370, 8400, 8465, 8490, 8540. *Neutral* 2190, 2480, 2570, 2840, 2880, 5120, 5390, 5500, 5510, 5532, 5534, 5731, 5740, 5800, 5900, 7000, 7002, 7004, 7006, 7009, 7010, 7025, 7030, 7034, 7035, 7040, 7060, 7080, 7090, 7100, 7140, 7150, 7175, 7190, 7217, 7224, 7233, 7235, 7491, 7950. *Unpleasant* 1300, 1301, 2053, 2120, 2710, 2800, 2900, 3160, 3220, 3230, 3300, 3350, 3500, 3530, 6200, 6210, 6212, 6230, 6244, 6250, 6260, 6312, 6313, 6370, 6540, 6550, 6560, 6570, 6571, 6821, 9040, 9050, 9421, 9490, 9520, 9600, 9620, 9911, 9920, 9921.
